# Translational control of Ybx1 expression regulates cardiac function in response to pressure overload in vivo

**DOI:** 10.1007/s00395-023-00996-1

**Published:** 2023-06-28

**Authors:** Eshita Varma, Jana Burghaus, Thomas Schwarzl, Thileepan Sekaran, Parul Gupta, Agnieszka A. Górska, Christoph Hofmann, Claudia Stroh, Lonny Jürgensen, Verena Kamuf-Schenk, Xue Li, Rebekka Medert, Florian Leuschner, Vivien Kmietczyk, Marc Freichel, Hugo A. Katus, Matthias W. Hentze, Norbert Frey, Mirko Völkers

**Affiliations:** 1 Department of Internal Medicine III (Cardiology, Angiology, and Pneumology), Heidelberg University Hospital, Im Neuenheimer Feld 410, 69120 Heidelberg, Germany; 2 DZHK (German Center for Cardiovascular Research), Partner Site Heidelberg/Mannheim, 69120 Heidelberg, Germany; 3 European Molecular Biology Laboratory, Meyerhofstrasse 1, 69117 Heidelberg, Germany; 4 Institute of Pharmacology, University Hospital Heidelberg, University of Heidelberg, Im Neuenheimer Feld 366, 69120 Heidelberg, Germany

**Keywords:** YBX1, mTOR, RNA-binding proteins, Heart failure

## Abstract

RNA–protein interactions are central to cardiac function, but how activity of individual RNA-binding protein is regulated through signaling cascades in cardiomyocytes during heart failure development is largely unknown. The mechanistic target of rapamycin kinase is a central signaling hub that controls mRNA translation in cardiomyocytes; however, a direct link between mTOR signaling and RNA-binding proteins in the heart has not been established. Integrative transcriptome and translatome analysis revealed mTOR dependent translational upregulation of the RNA binding protein Ybx1 during early pathological remodeling independent of mRNA levels. Ybx1 is necessary for pathological cardiomyocyte growth by regulating protein synthesis. To identify the molecular mechanisms how Ybx1 regulates cellular growth and protein synthesis, we identified mRNAs bound to Ybx1. We discovered that eucaryotic elongation factor 2 (Eef2) mRNA is bound to Ybx1, and its translation is upregulated during cardiac hypertrophy dependent on Ybx1 expression. Eef2 itself is sufficient to drive pathological growth by increasing global protein translation. Finally, Ybx1 depletion in vivo preserved heart function during pathological cardiac hypertrophy. Thus, activation of mTORC1 links pathological signaling cascades to altered gene expression regulation by activation of Ybx1 which in turn promotes translation through increased expression of Eef2.

**Supplementary Information:**

The online version contains supplementary material available at 10.1007/s00395-023-00996-1.

## Introduction

Heart failure (HF) is a detrimental clinical syndrome caused by either genetic or acquired heart diseases in which the cardiac output is insufficient to maintain the metabolic demands of the body*.* The high mortality makes HF a significant public health issue [35, 50]. As the underlying heart disease progresses into HF, heart size increases, cardiac function deteriorates, and symptoms of HF become evident. Although different terms have been used to describe it, the term *cardiac remodeling* encompasses many changes associated with progressive HF. Remodeling involves detrimental changes in cardiac structure, induced by either genetic or molecular alterations as well as stereotypical changes in gene expression independent from the underlying etiology.

Pathological gene expression was initially thought to be mainly regulated at the transcriptional level. However, studies analyzing only transcript levels ignore the significant contribution of post-transcriptional control mechanisms and, therefore, provide an incomplete picture of gene expression control in the heart [7, 11, 43, 47]. The unravelling of post-transcriptional mechanisms (e.g., mRNA processing, export, decay and turnover) delineated a complex regulatory network that controls organism and cell-type-specific gene expression patterns. Recently, we could show that mRNA transcript levels poorly correlate with levels of mRNA translation in the pathologically stressed heart, and a complex network of translationally regulated transcripts finetunes changes in gene expression in response to stress [11, 41].

Among several other mechanisms, RNA binding proteins (RBPs) have been identified as post-transcriptional regulators of gene expression [13, 19]. RBPs control tissue-specific gene expression by regulating splicing, mRNA stability, translation, and polyadenylation [14, 22, 28]. Previous studies have shown that specific RBPs such as RBM20 or PABC1 are crucial for cardiac function and adaptation in response to stress, but how expression or activation of individual RBPs is regulated in the diseased myocardium is largely unknown. The mammalian target of rapamycin complex 1 (mTORC1) is a major hub that controls diverse cellular processes [49], including the finetuning of gene expression by regulating mRNA translation, protein synthesis and degradation [4, 46]. Importantly, genetic, or pharmacological inhibition of mTORC1 is beneficial specifically for cardiomyocytes in heart failure, but a direct link between activity of specific RBPs in HF and mTORC1 signaling has not been comprehensively investigated.

We have used Ribosomal sequencing (Ribo-seq) and RNA sequencing (RNA-seq) to identify a subset of genes in cardiomyocytes that are regulated by mTORC1 [16] and have mapped the cardiomyocyte-specific RBPome using RNA interactome capture (RIC) [41]. Integrative analysis of those data identified a subset of RBPs in the heart that are translationally controlled by the activity of mTORC1 as well as during pressure overload induced by transverse aortic constriction (TAC).

Among them, Y box binding protein 1 (Ybx1) was strongly upregulated by mTORC1 signaling as well as after TAC. Ybx1 participates in the cellular response to stress and changes its cellular location, i.e., nucleus or cytoplasm, depending on the stimulus [29, 33]. Ybx1 regulates mRNA translation, depending on its concentration, RNA-partners, specific mRNAs or protein, and, possibly, mRNA modifications [39]. Previous in vitro studies in human and mouse cancer cells have shown that Ybx1 knockout reduces cancer cell proliferation [33, 39]. Moreover, a recent study showed that a cardiomyocyte-enriched circularRNA regulates cardiomyocyte proliferation and cardiac regeneration by post-transcriptional regulation of Ybx1 [20].

We hypothesized that Ybx1 is a critical cardiac RBPs for controlling gene expression and cardiac function in HF downstream of pathological mTORC1 signaling. To test our hypothesis, we characterized the function of Ybx1 in cardiomyocytes and showed that Ybx1 modulates pathological remodeling and cardiac function, both in vitro and in vitro. RNA-immunoprecipitation Sequencing (RIP-seq) against Ybx1 and Ribo-seq data after Ybx1 knockdown in cardiomyocytes were integrated and identified direct mRNA targets of Ybx1 in cardiomyocytes. Our results showed that Ybx1 depletion prevents pathological cardiomyocyte growth in vitro and promotes cardiac function in vivo by regulating protein synthesis, suggesting that the translationally controlled expression of Ybx1 is necessary to increase protein synthesis which promotes pathological cellular growth. Thus, our data further confirm the general importance of RBPs in gene expression in diseased cardiomyocytes and identified a specific regulatory network dependent on mTORC1 signaling that drives expression of Ybx1.

## Materials and methods

### *Isolation of neonatal rat ventricular cardiomyocytes *(*NRCMs*)

Neonatal rat cardiomyocytes (NRCMs) from 1 to 3-day-old Sprague–Dawley rats were prepared by trypsin digestion and percoll gradient separation following the standard methods [42]. Plastic petri dishes were coated with 0.1% gelatine for 1 h at 37 °C, and NRCMs were plated after removal of gelatine. NRCMs were plated in DMEM F12 with 10% FCS and 1% PSG. Cardiomyocytes were cultured at the following densities: 8 × 10^6^/15 cm dish,5 × 10^6^/10 cm dish 5 × 10^5^/6 well,3 × 10^5^/12 well.

### Isolation of adult mouse cardiomyocytes (ACM)

Adult cardiomyocytes from mice were isolated using the Langendorf-free method according to the protocol by Ackers-Johnson et al. [2]. In brief, after anesthesia of the 8-week-old Ythdf2 KO and WT mice, the heart was exposed by opening the chest. Following the cut of the descending aorta, injection of 7 mL EDTA containing buffer into the right ventricle and the clamping of the ascending aorta, the heart was transferred to a 60-mm dish with fresh EDTA containing buffer. For digestion 10 mL EDTA buffer, 3 mL perfusion buffer, and 30 to 50 mL collagenase buffer were injected into the LV sequentially. The LV was then separated from the other chambers and then gently pulled into 1-mm pieces using forceps. Cellular dissociation was completed by gentle trituration, and enzyme activity was inhibited by addition of 5 mL stop buffer. The cells were filtered through a 100-μm filter and restoration of calcium concentration to physiological levels was reached by several rounds of gravity settling, using 3 intermediate calcium reintroduction buffers. The highly pure cardiomyocyte population was plated in prewarmed plating media with around 50.000/cm^2^.

### siRNA induced knockdown

NRCMs were transfected with either 25 nM scrambled small interfering (siScr) RNA or siRNA targeting rat Ybx1 (siYbx1), after 24 h plating, using HiPerfect. The transfection solution was replaced with fresh DMEM F12 with 0.5% FCS and 1%PSG after overnight incubation. NRCMs were then cultured for 48 h before harvesting. Transfection experiments for gene expression analysis or western blotting were performed in duplicates or triplicates and repeated at least twice, otherwise indicated.

### Adenoviral overexpression in isolated NRCMs

To generate recombinant adenoviruses, the human Ybx1 and Eef2 cDNAs were subcloned into the pShuttle–CMV vector using the AdEasy XL Adenoviral Vector system (Stratagene) as previously described [16]. Normally stored frozen in viral storage buffer (20 nM Tris/HCL, 25 mM NaCl, 2,5% Glycerol, pH adjusted to 8.0 at 22 °C), the viral particles were added to the cell culture medium with 0.5% FBS with a multiplicity of infection (MOI, number infectious particles per cell) of 20. The cells were exposed to the viral medium for 24 h.

### Treatments

NRCMs were treated with the *α*_1_ adrenergic receptor agonist phenylephrine (PE) to mimic pathological cardiac hypertrophy. NRCMs were cultured in 10% FCS 1% PSG DMEM F12 for 24 h and starved for 48 h in 0.5% FCS 1% PSG DMEM F12. PE was freshly dissolved in PBS and added to the cells to obtain a final concentration of 50 µM. NRCMs were treated with PE for 24 h before harvesting for various experiments.

To inhibit mTOR pharmacologically, NRCMs were treated with 200 nM Torin1 for 3 h. Torin1 was prepared by dissolving it in DMSO and storing it at − 20 °C.

NRCMs were treated with 0.5 µgml puromycin for 30 min before being harvested.

A-484954 was used to inhibit Eef2 kinase in NRCMs. A-484954 (Eef2K inhibitor) was prepared by dissolving it in DMSO.NRCMs were cultured in 10% FCS 1% PSG DMEM F12 for 24 h and starved for 48 h in 0.5% FCS 1% PSG DMEM F12. NRCMs were treated with 100 uM final concentration of A-484954 for 24 h before harvesting.

### Immunoblotting

Cells were lysed in RIPA buffer, and protein determination was performed using Bio-Rad DC assay. Sampled were combined with Laemmli buffer and then heated at 95 °C for 5 min before SDS–PAGE followed by transfer to PVDF membranes. Antibodies concentrations as well catalog numbers are shown in table below:

**Table:** 

Antibody	Species	Dilution	Supplier	Catalog No
Actin	Mouse	1:1000	Santa cruz biotechnology	sc-8432
Eef2	Rabbit	1:5000	Cell signalling technology	2332S
GAPDH	Mouse	1:5000	Santa cruz biotechnology	sc-365062
Lamin B	Mouse	1:2000	Santa cruz biotechnology	Sc-374015
Peroxidase-AffiniPure Anti-Mouse	Donkey	1:5000	Jackson immuno research	715035151
Peroxidase-AffiniPure Anti-Rabbit	Goat	1:5000	Jackson immuno research	111035144
phospho-Eef2	Rabbit	1:1000	Cell signalling technology	2331
Phospho-p70 S6 Kinase	Rabbit	1:500	Cell signalling technology	9205L
Phospho-S6 Ribosomal Protein	Rabbit	1:5000	Cell signalling technology	4858S
Puromycin	Mouse	1:1000	Merck-Millipore	MABE343
S6 Ribosomal Protein	Music	1:1000	Cell signalling technology	54D2
Troponin T	Rabbit	1:2000	Abcam	ab209813
Ybx-1	Rabbit	1:2000	Cell signalling technology	D2A11

### RNA isolation and quantitative RT-PCR

Total RNA from NRCMs was isolated according to the manufacturer's protocol, with Quick-RNA™ MiniPrep (Zymo Research). Liquid nitrogen snap-frozen tissues were homogenized in Precellys 24 homogeniser (Bertin Instruments) in 500 ul of lysis buffer (RIPA or Mammalian Polysomal Lysis). A 100ul aliquot was mixed with 1 ml of Qiazol to isolate total RNA according to the standard protocols with chloroform and isopropanol precipitation. Quantitative RT-PCR was performed in triplicates on the samples using 3 μM of each primer and iTaq Universal SYBR Green Supermix. cDNA was created using the iScriptTM cDNA synthesis Kit (Bio-RAD). 18 s rRNA or HPRT were used to determine relative amounts of targets were determined using the ∆∆CT/nfold change method. Primers were designed using NCBI Primer BLAST and shown below.

### Primer used in the study

**Table:** 

Gene	Forward (5′–3')	Reverse (5′–3')
Rat Nppa	TACAGTGCGGTGTCCAACACAGAT	TGGGCTCAATCCTGTCAATCCTA
Rat Nppb	GAACAATCCATGATGCAGAAGC	GCTGTCTCTGAGCCATTTCCT
Rat Eef2	AAGTCCACGTTGACCGACTC	TGTCAGTGAAGCGTGTCTCC
Rat Ybx1	AAGTGATGGAGGGTGCTGAC	TGCCATCCTCTCTAGGCTGT
Rat Foxp1	ACGTGCCCATTTCTTCAGCAG	TGACGCACTGCATTCTTCCA
Rat Aldoa	CCCTCCTTACTCCTTTCGCC	CACAACACCACCCTTGGACT
Rat Ide	CGGTTCATCTCACTGGGTCC	ATACAACACGGGAGTGCAGA
Rat Acat	CATGGGCATCACAGCTGAAAAC	GCCCTTGATGACTGACTGGAT
Rat carns	TCGTCTTCTGATTGGTGAGGG	TCCAATGACACCTGCACACA
Rat, mouse HPRT	GGGGCTGTACTGCTTAACCAG	TCAGTCAACGGGGGACATAAA
Rat, mouse 18S	CGAGCCGCCTGGATACC	CATGGCCTCAGTTCCGAAAA
Mouse Nppa	TTGTGGTGTGTCACGCAGCT	TGTTCACCACGCCACAGTG
Mouse Nppb	TTTGGGCTGTAACGCACTG	CACTTCAAAGGTGGTCCCAGA
Mouse Ybx1	CAGGAGAGCAAGGTAGACCAGT	TGCTGACCTTGGGTCTCATCTC
Human Eef2	GACAGCGAGGACAAGGACAA	AGGCGTAGAACCGACCTTTG

### Silver staining

According to the manufacturer's protocol, silver staining was performed with the Pierce Silver stain kit (Thermo Fisher) to visualize proteins separated on SDS Polyacrylamide gels.

### Proximity ligation assay

Scrambled probe was designed for the assay based on previous publication [21].

Scrambled—A + CAC + TTAAC + CGTA + TAT + TCC + TA 21-mer with 6LNA.

OligoWalk algorithm of RNA structure 6.2 software was used for complementary ASO and for LNA modification ( +) Qiagen oligo optimiser was used.

Eef2.2390LNA—5′-TCGA + TGA + GGTT + GATG + AGG-3′.

Eef2. 2773LNA—5′-TGT + AGT + GCTG + AGTGA + TGT-3′.

The protocol for the proximity ligation assay (PLA) was followed according to a previously published work by Huppertz et. al. [15]. NRCMs were cultivated on gelatine-coated chamber slides and cultivated/treated like described above. Cells were fixed using 4% paraformaldehyde (PFA), permeabilized in PBS and blocked in PBS with 10% horse serum. NRCMs were the stained following the PLA protocol.

### Immunofluorescence and staining

Cell size measurements were done using immunofluorescence on NRCMs. NRCMs were cultivated on gelatine-coated chamber slides and cultivated/treated like described above. Cells were fixed using 4% paraformaldehyde (PFA), permeabilized in PBS and blocked in PBS with 10% horse serum. Primary antibodies were diluted in 10% horse serum for overnight incubation at 4 °C. The subsequent day, cells were washed with PBS and incubated for 1 h at RT with 1:5000 dilution of secondary antibody (Jackson Laboratories). Cells were mounted in mounting media supplemented with 1:10,000 DAPI as nuclear staining. For cell size analysis, 20 × and 63 × pictures were taken using the Zeiss Axio Observer.Z1 fluorescence microscope.

**Table:** 

Antibody	Species	Dilution	Supplier	Catalog No.
Anti-Biotin	Mouse	1:750	AbCam	ab201341
Anti-Biotin	Rabbit	1:750	AbCam	Ab234284
Anti-rabbit IgG-FITC	Donkey	1:100	Jackson Immuno Research	711-095-152
Anti-rabbit IgG-Cy3	Donkey	1:100	Jackson Immuno Research	711-165-152
Anti-Mouse IgG-FITC	Donkey	1:100	Jackson Immuno Research	715-095-151
Anti-Mouse IgG-Cy3	Donkey	1:100	Jackson Immuno Research	715-165-151
Eef2	Rabbit	1:200	Cell Signalling Technology	2332S
Sarcomeric Actin	Mouse	1:100	Santa Cruz Biotechnology	sc-8432
Ybx-1	Rabbit	1:200	Cell Signalling Technology	D2A11
*Ki67*	*Rabbit*	*1:50*	*Proteintech*	*27,309-1-AP*

### Cell size measurement

Cell size assessment was completed using Fiji software (https://imagej.net/software/fiji/). The measurements of the cell size were performed manually. We conducted image measurement analysis on ImageJ (Fiji) using a blinded approach, where we were unaware of the treatment allocation for each sample. The images were imported without any identifying information, and anonymous labels were assigned to each sample. Randomized measurements were performed to avoid biases, and the results were compiled and analyzed without revealing the treatment allocation, ensuring an unbiased analysis. At least 60 cardiomyocytes were analyzed from every experiment. Cells were stained with Ybx1, alpha sarcomeric actin and DAPI.

### Quantification of proliferation

To detect EdU incorporation, cells were stained using a Click-it EdU Imaging Kit (Life Technologies, #C10638) according to the manufacturer’s instruction. NRCMs were incubated for 8 h with 3um EdU in 5% Serum. Cells were fixed using 4% paraformaldehyde (PFA), permeabilized in PBS and EdU incorporation detected using Click-Chemistry. NRCMs were detected by staining with sarcomeric actin and EdU positive NRCMs visualized and using a Zeiss Axio Observer.Z1 fluorescence microscope. In parallel, NRCMs were stained for the nuclear antigen Ki67 and Ki67 positive cells were counted and quantified.

### Complex capture

NRCMs were plated in 15 cm dishes with 10% FCS 1% PSG DMEM-F12 and then UV cross-link according to the protocol. Cells were harvested in Mammalian Polysomal buffer and protein determination was performed using Bio Rad Assay. RNA isolation was performed using the Zymo Quick RNA Isolation kit. 2 mg of protein sample (X) was mixed with 4X lysis buffer and 5X 100% ethanol. RNA isolation was performed according to the Bio-Rad kit and it was finally eluted in 20 ul nuclease free water. RNA concentration was measured on Nano drop and then samples were treated with or without RNase. Samples were then used for silver staining or western blot.

### RNA-immunoprecipitation

NRCMs were harvested, after cultivation in DMEM-F12 containing 10% FCS for 24 h and in DMEM-F12 in 0.5% FCS for 47 h, in ice cold mammalian polysome buffer (10 mM MgCl, 20 mM Tris pH 7.4, 2 mM DTT, 200 mM KCl, 1% Triton X-100,) containing 40 U/µl Murine RNAse inhibitor, and1 x protease inhibitor cocktail and sonicated for complete lysis. Lysate was incubated with Ybx1 antibody (D2A11) overnight at 4 °C, then for 1 h at RT with prewashed sheep anti rabbit IgG Dynabeads (ThermoFisher). Coprecipitate was washed once with ice cold wash buffer (polysomal buffer with 10% Triton, DNase I), three times with ice cold high salt buffer (polysomal buffer with 1 M KCl, 10% Triton, DNase I) and then once finally with wash buffer. Samples were divided for use for protein or RNA. Protein elution was performed by denaturing the samples with laemmli buffer at 95 °C for 5 min.RNA was eluted using Trizol and following extraction using chloroform. Library generation was done using the Lexogen Quant-Seq kit according to the manufacture’s instruction. Libraries were then multiplexed and sequenced on a NextSeq550.

### Polysome profiling and Ribo-seq

Two biological replicates were used for creating Ribo-seq libraries. Ribosomal footprints were generated after isolation of Polyribosomes from sample lysates and RNAse I digestion as previously published [20]. Briefly, 1 15-cm dish of NRCMs was lysed in 500 µl polysome buffer (20 mM Tris pH 7.4, 200 mM KCl, 10 mM MgCl, 2 mM DTT, 100 μg/ml CHX, 1% Triton X-100, 1U DNAse/μl). The cell lysates were lysed on ice for 10 min and then centrifuged at 20,000×*g* to precipitate cell debris. The supernatant was immediately separated and then used in the following steps. Sucrose gradient was created using 10% and 50% sucrose solutions prepared in ice cold sucrose buffer containing 10 mM Tris–HCl pH 8.0, 5 mM MgCl_2_, 100 mM KCl, 0.1 mg/ml CHX, 1 × Protease inhibitor cocktail and 20 U/mL SUPERase-In. The ribosomal libraries were generated and sequenced following previously published protocol [42].

### shRNA knockdown in vivo

AAV9 was generated using shRNAs plasmid against Ybx1 for in vivo administration. To achieve a specific reduction of Ybx1 protein expression with the help of an RNAi strategy, two different commercially available microRNA-30-based shRNAs (shYbx1) targeting murine Ybx1 were chosen (Vigene Biosciences). A non-effective scrambled shRNA was used in the control vector. We PCR amplified the shRNA miR30 sequences (Scramble or shYbx1) and cloned it into a self-complementary AAV plasmid under a CMV promoter and enhancer in the plasmid. Recombinant AAV9 (rAAV9) vector particles were generated and purified using the iodixanol gradient ultracentrifugation method [16]. For the RNAi-based reduction of Ybx1, high cardiac transduction efficiency as well as a high AAV9 copy number per cardiomyocyte was achieved by the application of 3 × 10^12^ vg/mouse. The sequence for Ybx1 insert in the plasmid was as follows:

Ybx1-

CGCCATGAGCAGCGAGGCCGAGACCCAGCAGCCGCCCGCCGCCCCCCCCGCCGCCCCCGCCCTCAGCGCCGCCGACACCAAGCCCGGCACTACGGGCAGCGgcgcagggAGCGGTGGCCCGGGCGGCCTCACATCGGCGGCGCCTGCCGGCGGGGACAAGAAGGTCATCGCAACGAAGGTTTTGGGAACAGTAAAATGGTTCAATGTAAGGAACGGATATGGTTTCATCAACAGGAATGACACCAAGGAAGATGTATTTGTACACCAGACTGCCATAAAGAAGAATAACCCCAGGAAGTACCTTCGCAGTGTAGGAGATGGAGAGACTGTGGAGTTTGATGTTGTTGAAGGAGAAAAGGGTGCGGAGGCAGCAAATGTTACAGGTCCTGGTGGTGTTCCAGTTCAAGGCAGTAAATATGCAGCAGACCGTAACCATTATAGACGCTATCCACGTCGTAGGGGTCCTCCACGCAATTACCAGCAAAATTACCAGAATAGTGAGAGTGGGGAAAAGAACGAGGGATCGGAGAGTGCTCCCGAAGGCCAGGCCCAACAACGCCGACCCTACCGCAGGCGAAGGTTCCCACCTTACTACATGCGGAGACCCTATGGGCGTCGACCACAGTATTCCAACCCTCCTGTGCAGGGAGAAGTGATGGAGGGTGCTGACAACCAGGGTGCAGGAGAACAAGGTAGACCAGTGAGGCAGAATATGTATCGGGGATATAGACCACGATTCCGCAGGGGCCCTCCTCGCCAAAGACAGCCTAGAGAGGACGGCAATGAAGAAGATAAAGAAAATCAAGGAGATGAGACCCAAGGTCAGCAGCCACCTCAACGTCGGTACCGCCGCAACTTCAATTACCGACGCAGACGCCCAGAAAACCCTAAACCACAAGATGGCAAAGAGACAAAAGCAGCCGATCCACCAGCTGAGAATTCGTCCGCTCCCGAGGCTGAGCAGGGCggggctgaga.

2-month-old mice were injected with AAV9, and then 3 weeks after injection, TAC surgeries were performed on the mice. TAC surgeries were performed according to our previous work [42]. 2 weeks after TAC surgery, mice were sacrificed, and hearts were collected by snap freezing in liquid nitrogen.

Mice left ventricles were snap-frozen hearts and lysed in lysis buffer (1 × Mammalian polysome buffer, 1% Triton, 1 × phosphatase inhibitor, 1 × protease inhibitor) or RIPA buffer (12.5 mM Tris pH 7.5, 75 mM NaCl, 0.05% SDS, 0.5% Triton X, 0.5% Na-deoxycholate, 1 × protease inhibitor, 1 × phosphatase inhibitor) using a magnetic bead mill. Lysates were diluted in RIPA buffer for western blot. For RNA isolation, Qiazol was added to the samples, and chloroform was used for extraction using the clean concentrator kit (Bio-Rad).

### Echocardiography

Visual Sonics Vevo 2100 Imaging System was used to perform the echocardiography. Images were taken with the MS-M550D transducer from the parasternal long and short heart axis to characterize anterior and posterior wall thickness and function. First, images were taken in B-mode, followed by a time-dependent cross section in M-mode.

Animals were shaved before measurements to enable better picture quality with less background. Animals were then sedated with 2.5 vol% Isoflurane and fixed in a horizontal position onto the heated plate (37 °C). To achieve clear transmission of heart and breathing rates electrode gel was used. Images were recorded at a heart rate between 400 and 450 beats per minute to allow comparison between images and avoid bias in heart function measurements.

The evaluation was accomplished using the standard 2D quantification software.

### RIP-Seq analysis

Reads were trimmed with cutadapt (v2.5) and mapped to rat genome (Rnor_6.0) with STAR (v2.7) and summarized with featureCounts (v1.6.4). DESeq2 with IHW for multiple hypothesis correction was used to determine significantly enriched RNAs in IP samples vs. corresponding input controls (adjusted *p* value < 0.1; log_2_ fold-change > 0.5).

### RiboSeq analysis

To investigate the RiboSeq libraries we utilized our previously published protocols [9]. Adapters were removed with Flexbar v3.0.3 using standard filtering parameters. Reads were aligned to a custom bowtie2 v2.3.0 ribosomal index were discarded. The residual reads were then aligned to Rnor_6.0 with STAR. For RiboSeq data, only periodic fragment lengths that indicated a specific triplet periodicity were kept. For the statistical analyses, we use the edgeR package. We filtered for data points with read count observations across all replicates. We used a cutoff of an adjusted *p* value < 0.05 and fold change < 2.

### Statistics

In vivo experiments were performed on 4–20 biological replicates (mice) and the in vitro experiments were performed with at least 3 biological replicates for each treatment. Throughout the studies, the investigators were blinded to the sample group allocation during the experiment and analysis of the experimental outcome. Statistical analysis was performed using GraphPad Prism 7.0 (Graphpad Software Inc; www.graphpad.com) or R. All the data sets were tested for normality of distribution using the Shapiro–Wilks test (threshold *P* < 0.05). For normally distributed data, values shown are mean ± SEM. Statistical analysis of data involving two groups was performed using unpaired two-tailed *t* test, for more than two groups one-way ANOVA with the Bonferroni test applied to correct for multiple comparisons. Sequencing count data were modelled using negative-binomial distribution. For not normally distributed data a nonparametric test was used to test for significance between different groups. A Mann–Whitney test was performed when comparing two groups. A Kruskal–Wallis test was used when comparing multiple groups (more than two) followed by a Dunn's multiple test comparison.

## Results

### Ybx1 is translationally upregulated by mTOR

**Fig. 1:**
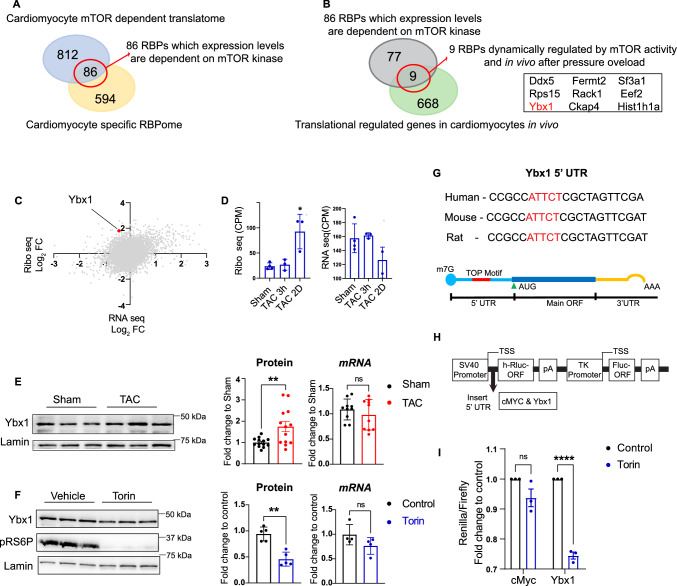
Ybx1 is translationally upregulated by mTOR during cardiac hypertrophy. (**A**) Venn Diagram showing the overlap of mTORC1 responsive mRNAs and RBPs in NRCMs. (**B**) The 86 hits were then overlapped with mRNAs upregulated 2 days after TAC in vitro, resulting in 9 candidate RBPs. (**C**) Scatter plot of Ribo Seq and RNA seq data 2 days after TAC. Ybx1 is highlighted in red. (**D**) Quantification of Ybx1 in Ribo-Seq and RNA-Seq data after sham, or TAC surgery for indicated timepoints. (**E**) Representatives immunoblots and quantification of Ybx1 protein levels 2 days after TAC and Sham in vivo*.* Error bars indicate means ± SEM *n* = 13 *p ≤ 0.05 vs. Sham surgery, ^#^*p* ≤ 0.05 vs. TAC 2d surgery, *t* test (**F**) Representative immunoblot and quantification of Ybx1 expression levels in NRCMs after inhibition by Torin1 for 3 h. Lamin B is used as a housekeeping protein. *n* = 5 independent experiments (**G**) Highly conserved 5' UTR sequence for Ybx1 in human, mouse, and rat. The TOP-like motifs are highlighted in red. (**H**) Schematic representation of reporter consisting of human Ybx-1 or Eef2 or c-Myc 5’ UTR sequence. (**I**) Dual luciferase assay measurements with different 5' UTR regions. Luciferase activity was measured in normal conditions and after mTORC1 inhibition with Torin1 for 3 h. Eef2—positive control of reporter with defined 5’TOP motif and non-TOP 5’ UTR of cMyc was used a negative control. *t* test with Bonferroni–Dunn correction, *n* = 3. Error bars indicate means ± SEM ***p ≤ 0.001, *p* ≤ 0.0001

We identified 86 RBPs translationally regulated by the mTOR pathway in cardiomyocytes by integrating data sets that have (1) identified translationally controlled mRNAs after treatment with the mTOR kinase inhibitor Torin in vitro by Ribo-seq [16] and (2) defined cardiomyocyte-specific RBPs identified via RNA interactome capture (RIC) [41] (Fig. 1A). Those previously generated data sets helped us to identify RBPs that are controlled by mTORC1 signaling [11, 16, 41]. We integrated these 86 RBPs with an in vivo Ribo-Seq data set [11] that used the Ribo-tag mice [42] after transverse aortic constriction (TAC) with the goal to filter for RBPs that are also regulated in vivo in cardiomyocytes (Fig. 1B). The overlap of the 86 RBPs with the in vivo data identified nine RBPs that are (1) dynamically regulated on the translational level during acute pressure overload and (2) translational controlled by mTOR activity. The role of Ybx1, one of the nine implicated RBPs, in the heart in vivo was largely unknown, but Ybx1 has been recently shown to regulate NRCM proliferation [20]. Therefore, we decided to characterize the role of Ybx1 during cardiac remodeling. Ribo-seq suggests a translational upregulation of Ybx1 during cardiac hypertrophy, while *Ybx1* mRNA levels remain virtually unchanged according to RNA-seq (Fig. 1C, D). Immunoblot analysis confirmed that Ybx1 protein levels are upregulated 2 days after TAC surgeries independent from mRNA levels assessed by RT-qPCRs (Fig. 1E). To confirm the translational dependence of Ybx1 on mTOR, we treated NRCMs with Torin1. Western blot and RT-qPCR in NRCMs confirmed the translational mTOR dependency of Ybx1 expression in response to Torin1 (Fig. 1F). While Ybx1 was downregulated on the protein level after the cells were treated with 150 nM Torin1 for 3 h, mRNA levels are unchanged, suggesting a correlation between Ybx1 and mTOR signaling in cardiomyocytes.

The mTOR pathway is critically involved in the regulation of mRNA translation, and a 5' Terminal Oligo Pyrimidine (5' TOP) motif is often present in mTOR responsive mRNAs that encode for proteins required for protein synthesis. Studies have highlighted the importance of the mTORC1 signaling pathway in regulating TOP mRNAs [8] and mRNAs containing a 5' TOP-like motif are sensitive to mTOR inhibitors, such as rapamycin and Torin1 [40]. Ybx1 has a highly conserved 5' UTR region, and a TOP-like motif is also highly conserved between species (Fig. 1G). To investigate the sensitivity of the TOP-like motif in the 5'UTR region of Ybx1 to mTOR, we used a dual-luciferase reporter vector with renilla and firefly luciferase. We cloned the human 5' UTR region of Ybx1 or c-Myc mRNA upstream of renilla luciferase, the firefly luciferase was used as control, and the renilla/firefly ratio determines the dependence on mTOR (Fig. 1H). The Ybx1 5' UTR showed a strong reporter inhibition after Torin1 treatment compared to the control luciferase construct (Fig. 1I). This suggests mTORC1-dependent translation of Ybx1 as well as the presence of a regulatory motif in its 5' UTR. Collectively, our results indicate that mTORC1 post-transcriptionally regulates Ybx1 expression in response to pathological stimulation.

### Ybx1 is an RNA binding protein in cardiomyocytes

**Fig. 2:**
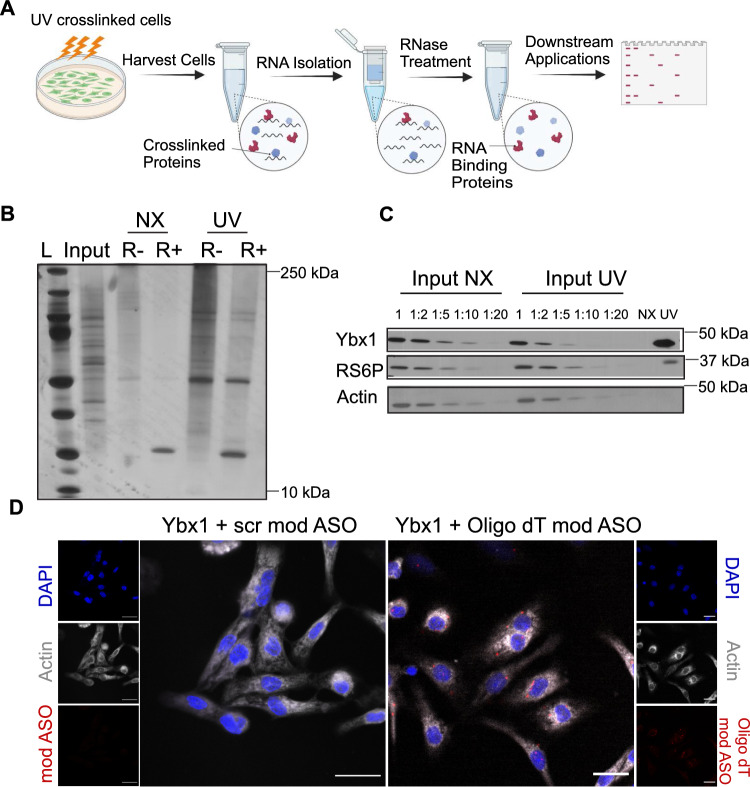
Ybx1 binds to RNA in cardiomyocytes. (**A**) Experimental design of complex capture protocol. Cells were irradiated with UV and then protein–RNA complexes were isolated using RNA-affinity columns. Unbound RNA was digested with RNases and complexes were visualized by silver staining or Western Blot. The schematic illustration was created with Biorender.com (**B**) Silver Gel of NRCMs samples that were non-crosslinked (NX) or cross-linked (UV) and then treated with (+) or without (−) RNase. (**C**) Western blot of NRCMs lysates after complex capture. Inputs were diluted in various concentrations and the samples were treated with RNAse before loading them on the gel. The presence of Ybx1 in the UV crosslinked and the absence of the Ybx1 in the non-crosslinked (NX) confirms Ybx-1 as an RBP. Ribosomal S6 Protein (RS6P) was used a positive control and Actin was used as a negative control. (**D**) Representative immunofluorescence image of a proximity ligation assay (PLA) for Ybx1 with either scrambled modASO or oligo dt modASO (red dots). The interaction between Ybx1 and modASO is detected using anti-Biotin antibody. The sarcomeric actin is shown in grey and the blue highlights the DAPI staining. The scale bar represents 20 µms

To confirm that Ybx1 binds to RNA in NRCMs, we performed a technique called complex capture [3]. NRCMs were harvested after UV cross-linking, and RNA–protein complexes were isolated using a Zymo RNA kit. Isolated total RNA was treated with and without RNase to digest all the RNA, leaving only protein in the final sample (Fig. 2A). The RNA was loaded onto a polyacrylamide gel, and silver staining was performed to detect enriched proteins after UV-crosslinking of RNA with proteins. While no proteins were detected in the non-crosslinked sample, specific protein bands were observed in the cross-linked samples treated with RNase (Fig. 2B). After UV-crosslinking, immunoblotting for Ybx1 in the eluates confirmed Ybx1 as an RBP in cardiomyocytes (Fig. 2C). In addition, we confirmed the binding of Ybx1 with mRNAs using an immunofluorescence-based, UV crosslinking-independent RNA proximity ligation assay (PLA). This PLA detects endogenous or tagged proteins with their RNA targets in situ [21]. Modified antisense oligonucleotide (modASO) scrambled or complementary to the poly A tail (oligo dT) was used as negative or positive control, respectively. Interaction of Ybx1 with the oligo dT modASOs shows that Ybx1 binds to mRNAs in NRCMs (Fig. 2D).

### Change in Ybx1 reduces cell size and protein translation

**Fig. 3:**
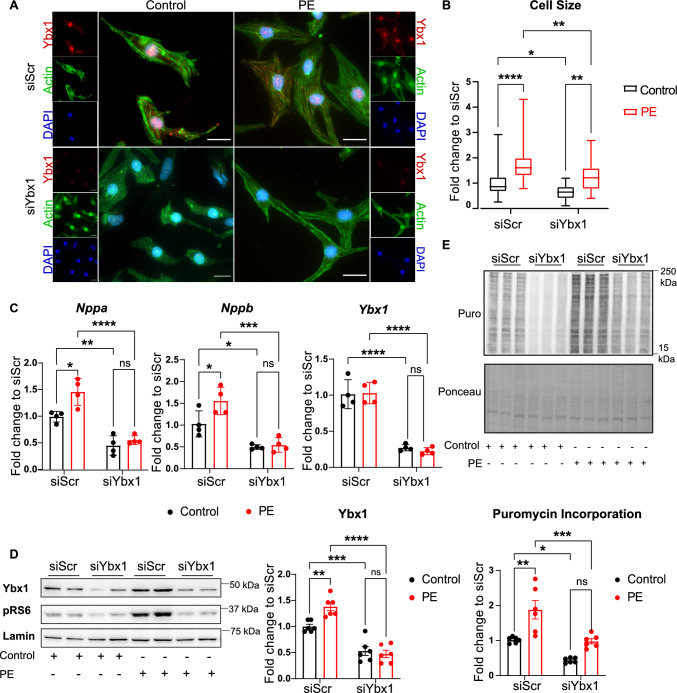
Ybx1 knockdown prevents cellular growth in vitro by inhibiting protein translation. (**A**) Immunofluorescence staining of NRCMs for Ybx1 (red), sarcomeric actin (green) and nuclei (blue) after knockdown of Ybx1 (siYbx1) or scramble (siScr) with/without PE (50 µM) treatment for 24 h. Scale bar 20 μm. (**B**) Quantification of cell size measurements. Analyzed by One-way ANOVA. *n* > 150 cells from *n* = 3 independent experiments. (**C**) Quantitative PCR for expression levels of *Nppa*, *Nppb* and *Ybx1* in NRCMs to confirm the knockdown and the PE treatment. (**D**) Representative immunoblots and quantification of Ybx1 protein levels in NRCMs after knockdown of Ybx1 and stimulation with PE. Phospho ribosomal S6 protein (pRS6) is used as a marker for the effect of PE treatment. Lamin B is used as a control. *n* = 6 independent experiments (**E**) Representative immunoblot and quantification of Puromycin incorporation in NRCMs after knockdown with scrambled (siScr) or Ybx1 (siYbx1), followed by PE treatment. Ponceau staining was used as a gel loading control. *n* = 4–6 per group. *t* test, *P ≤ 0.05, **P ≤ 0.01, ****P ≤ 0.0001. Error bars indicate mean ± SEM

Previous research in cancer cells has indicated that depletion of Ybx1 reduces cell growth and proliferation [27, 30]. The effect of Ybx1 on cardiomyocytes was analyzed by knocking down Ybx1 using siRNA. To mimic pathological cardiomyocyte growth, NRCMs were treated with the α-1 receptor agonist phenylephrine (PE) for 24 h. The effect of Ybx1 on the cell size of NRCMs was evaluated by immunostaining after the knockdown. RT-PCR analysis showed that Ybx1 knockdown with siRNA resulted in an approximately 80% reduction in Ybx1 mRNA levels. PE treatment resulted in a 50% increase in the cell surface area compared to vehicle-treated cells in NRCMs transfected with a control siRNA. However, Ybx1 knockdown resulted in a significant reduction in cell size under both baseline conditions, and after neurohumoral stimulation with PE for 24 h (Fig. 3A, B).

Similarly, PE induced the expression of the hypertrophy marker genes *Nppa* and *Nppb*, quantified by RT-qPCR, but the induction was blocked after the siRNA-mediated knockdown of Ybx1. PE caused a 1.5-fold increase in *Nppa* and *Nppb* levels compared to the vehicle-treated control. Ybx1 knockdown caused a 50% reduction in *Nppa* and *Nppb* levels in both vehicle and PE-treated samples (Fig. 3C). Immunoblotting confirmed upregulation of Ybx1 after PE stimulation in siScr transfected cells and successful knockdown of Ybx1 in the siYbx1 transfected cells, while mRNA levels remain unchanged (Fig. 3D). To analyze the effect of Ybx1 on the overall protein translation in NRCMs, a puromycin assay was performed after PE treatment and knockdown of Ybx1. A reduction in puromycin incorporation levels was observed for NRCMs with Ybx1 knockdown (Fig. 3E), suggesting that Ybx1 knockdown reduces protein translation in NCRMs at baseline. Moreover, stimulation of NRCMs with PE resulted in almost a twofold increase in overall protein synthesis, but this increase was completely blocked with the knockdown of Ybx1.

We also aimed to characterize the effect of Ybx1 overexpression in NRCMs. Ybx1 was overexpressed in NRCMs using adenoviral vectors. Cell size and protein translation were evaluated 24 h after Ybx1 overexpression using immunostaining and puromycin incorporation, respectively. Interestingly, Ybx1 overexpression in NRCMs resulted in a reduction of cell size that was associated with reduced puromycin incorporation (Supplementary Fig. 1A–D), independent from mTORC1 signaling. Those experiments archived an twofold increase in Ybx1 protein expression level (Supplementary Fig. 1E), similar to levels observed after neurohumoral stimulation. Those data indicate that, while Ybx1 is required for cellular growth, Ybx1 levels must be tightly controlled and increased Ybx1 levels itself might counterbalance the cellular growth response by still not fully understood mechanisms.

### Identification of Ybx1 mRNA targets

**Fig. 4:**
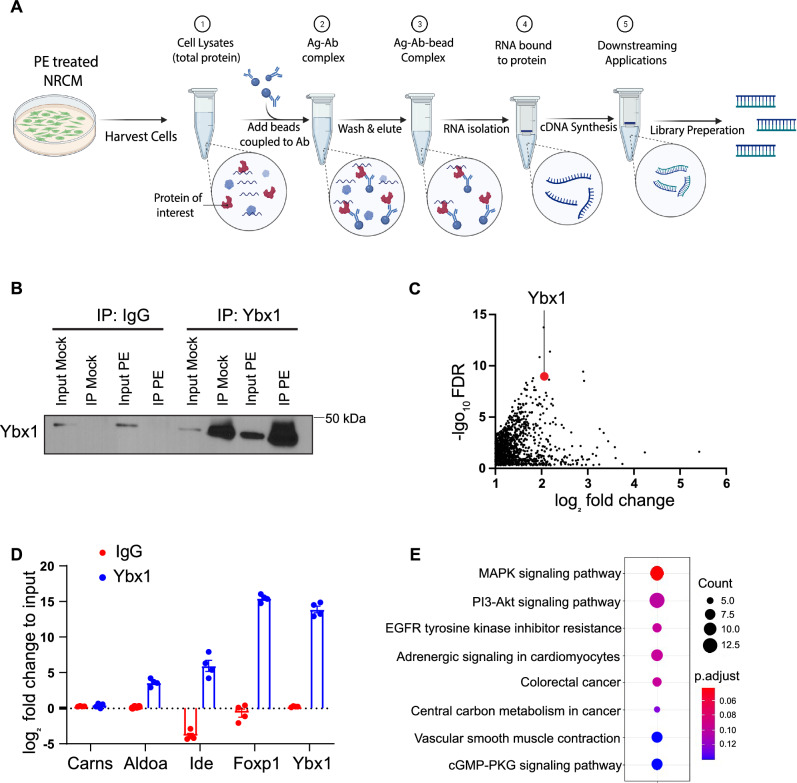
Identification of mRNAs binding to Ybx-1 in NRCMs. (**A**) Experimental design for RNA Immunoprecipitation (RIP)-Seq in NRCMs. NRCMs were treated with PE (50uM) for 24 h and then harvested. Beads and antibody were incubated at room temperature for an hour and then the cell lysate was added to the mixture. The protein (Ag)-antibody (Ab) bead mixture was incubated overnight and then washed to remove any unbound protein from the beads. Finally, RNA was isolated and used for RNA-Seq. Illustration created with Biorender.com (**B**) Immunoblot showing the binding of Ybx1 after immunoprecipitation. (**C**) Scatter plot of the mRNAs bound to Ybx1 identified by RIP-seq in NRCMs. Full data set can be found in Supplementary Table 1. Ybx1 mRNA is known to bind its own transcript and is, therefore, highlighted in red. (**D**) Validation of the hits from the RIP-Seq by RIP–RT–PCR. Ybx1, Foxp1, Aldoa, Ide were taken as positive controls and Carns was used as negative control. (**E**) KEGG pathway enrichment for the mRNAs bound to Ybx1

To identify mRNAs that are bound to Ybx1, we performed RNA-immunoprecipitation followed by high throughput sequencing (RIP-Seq) in NRCMs (Fig. 4A). Immunoblots confirmed specific Ybx1 immunoprecipitation (Fig. 4B). RNA was isolated from the cell input lysates after Ybx1 IP and analyzed by RNA-Seq. Among 12,310 transcripts in the input, 5.7% (707) were bound to Ybx1 with a fold change greater than 1 and FDR less than 0.05. *Ybx1* mRNA binds to Ybx1 protein to self-regulate its expression [30]; therefore, the presence of the Ybx1 mRNA was used as a positive control for the RIP-Seq data (Fig. 4C). We performed RIP-PCR to validate the hits from the RIP-Seq data. Since, *Ybx1, Acta1, Foxp1, Aldoa, and Ide* were reported to be Ybx1 target mRNAs; we used primers against these transcripts as positive control and *Acat, Carns* were used as negative control (Fig. 4D). Among Ybx1-bound RNAs, we found significant enrichment of transcripts encoding for mRNA involved in signaling pathways, such as MAPK and PI3-Kinase signaling (Fig. 4E).

**Fig. 5:**
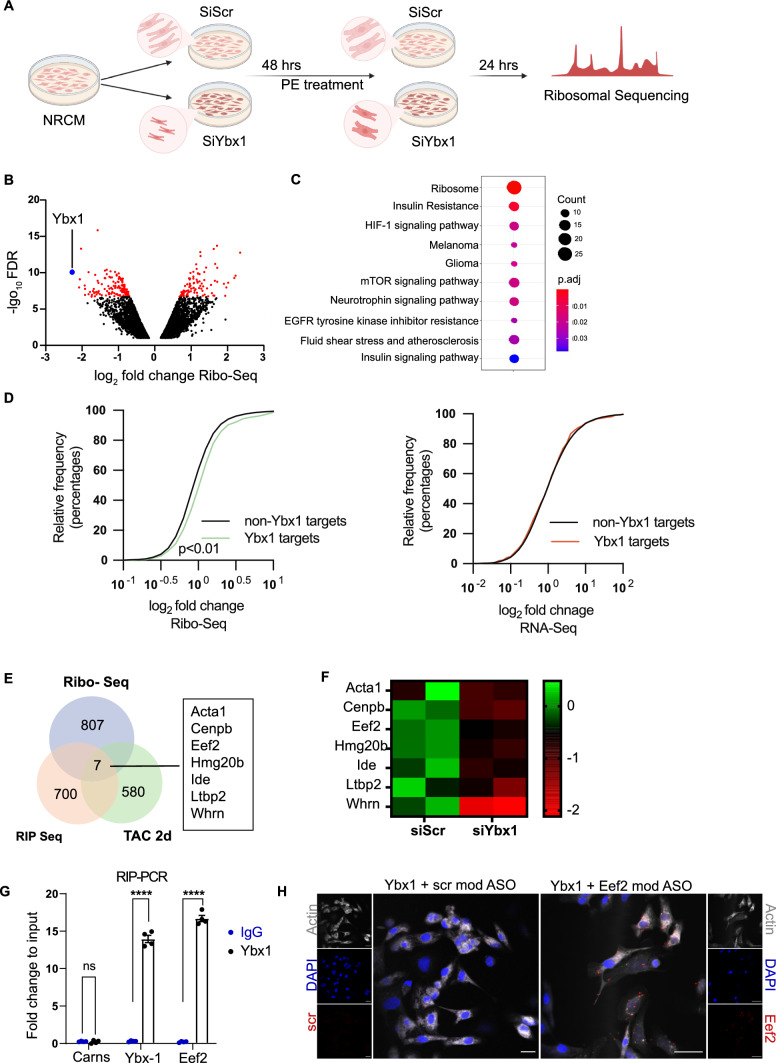
Identifying transcripts regulated by Ybx-1. (**A**) Experimental design of ribosomal profiling in NRCMs. Ybx1 was knocked down using siRNA, after 48 h NRCMs were treated with PE for 24 h and then harvested for ribosomal profiling. Illustration created with Biorender.com. (**B**) Scatterplot representing genes upregulated and downregulated due to knockdown of Ybx1 in NRCMs. The knockdown of Ybx1 is highlighted in blue. Significant regulated genes are highlighted in red. Full data set can be found in Supplementary Table 2. (**C**) KEGG pathway enrichment of proteins affected by Ybx-1 knockdown. (**D**) Cumulative fraction of mRNAs relative to their fold change of Ribo-seq (Kolmogorov–Smirnov test *p* < 0.01) or RNA-seq between all transcripts and Ybx-1 targets identified by RIP-seq 2 days after TAC surgery. (**E**) Venn diagram showing the overlap of the mRNAs affected by Ybx1 knockdown (Ribo-Seq), upregulated 2 days after TAC (Ribo-Seq) and bound to Ybx-1 (RIP-Seq). (**F**) Heatmap of 7 common targets and the difference in expression levels in scrambled and Ybx-1 knockdown. (**G**) RIP-PCR for validation of Eef2 mRNA binding to Ybx-1. Carns was used as a negative control. (**H**) Representative image of a proximity ligation assay (PLA) for Ybx-1 with either scrambled or Eef2 modASO (red dots). The sarcomeric actinin is shown in grey and the blue highlights the DAPI staining. The scale bar represents 20 µms

Next, we sequenced actively translated mRNAs by Ribo-seq in NRCMs after siRNA-mediated knockdown of Ybx1 compared to control NRCMs (Fig. 5A). Ribo-seq analysis identified in total 9638 translated transcripts in NRCMs. Ybx1 was the transcript with the highest reduction as compared to the scramble (Fig. 5B) and we identified 942 (9.7%) transcripts that were differentially translated after reduction in Ybx1 levels in NRCMs. Analysis of the significant Ribo-seq hits, highlighted the ribosome as well as the mTOR pathway as one of the GO-terms enriched in the KEGG pathway (Fig. 5C). Globally, Ybx1 targets were highly translated in response to TAC surgery (Fig. 5D) compared to sham. In contrast, fold changes of transcript levels of Ybx1 targets were similar to non-Ybx1 targets, suggesting that Ybx1 binding to mRNA is associated with increased translational efficiency in response to TAC in vivo.

To focus on mRNA targets affected by Ybx1 knockdown and bound to Ybx1, we integrated the Ribo-Seq and RIP-Seq data. Parallel integration of Ribo-Seq results in vivo 2 days after TAC and in vitro in NRCMs after 24 h PE treatment as well as the RIP-seq data emerged 7 final common targets between the in vitro and in vivo data sets, which are (1) bound to Ybx1, (2) translated dependent on Ybx1 and (3) translationally regulated after in vitro TAC surgery in cardiomyocytes (Fig. 5E, F).

Eef2 is a GTP-binding protein that is an essential factor for protein synthesis that controls the translocation of peptidyl-tRNA from the A site to the P site on the ribosome; however, its specific role in the pathological remodeling of the heart is largely unknown. Therefore, for validation of the RIP-seq, we quantified Eef2 transcript levels in the RIP samples from PE-treated NRCMs. Indeed, *Eef2* mRNA is bound by Ybx1. Primers against *Ybx1* were used as a positive control, and *Carns* was used as a negative control (Fig. 5G). Ybx1 binding to target mRNAs was also confirmed using modASO complementary to Eef2 mRNA, which showed the interaction between Ybx1 and Eef2 in NRCMs (highlighted in red in Fig. 5H).

### Ybx1 increases Eef2 translation during myocardial hypertrophy

**Fig. 6:**
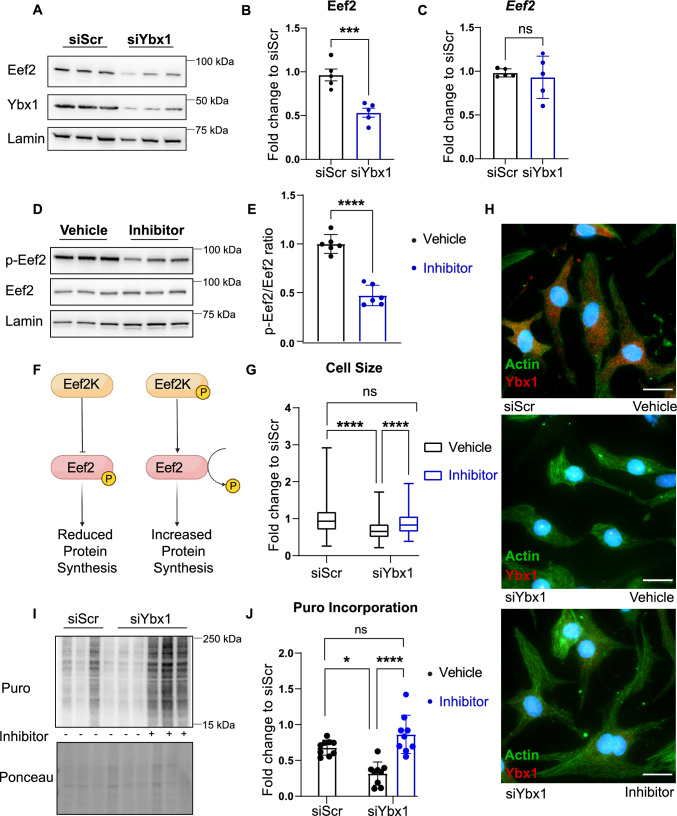
Eef2K inhibitor increases cell size and protein translation in NRCMs. (**A**) Representative immunoblots of Ybx1 knockdown in NRCMs highlighting the reduction in Eef2 protein levels along with the reduction in Ybx1 protein levels. Lamin B was used as a housekeeping protein. (**B**) Quantification of Eef2 protein levels in NRCMs after Ybx-1 knockdown. (**C**) Quantification of Eef2 mRNA levels after Ybx1 knockdown in NRCMs normalized to HPRT (**D**) Representative immunoblot of NRCMs treated with and without A-484954, an Eef2K inhibitor (inhibitor) for 24 h. Lamin B was used as a housekeeping protein. (**E**) Quantification of phosphorylation of Eef2 after treatment with A-484954 (inhibitor) for 24 h. (**F**) Schematic diagram of Eef2K and Eef2 affecting overall protein synthesis. (**G**) Quantification of cell size measurements. Analyzed by One-way ANOVA. *n* > 150 cells from *n* = 3 independent experiments. (**H**) Immunofluorescence staining of neonatal rat cardiomyocytes for Ybx1 (red), sarcomeric actin (green) and nuclei (blue) after knockdown of Ybx1 or scramble and with/without Eef2 Kinase inhibitor (100 uM). Scale bar 20 μm. (**I**) Representative immunoblots of puromycin incorporation in NRCMs treated with Eef2 inhibitor for 24 h after Ybx1 knockdown. (**J**) Quantification of Puromycin incorporation in NRCMs after Ybx1 knockdown and treatment with Eef2K inhibitor. One-way ANOVA, *n* = 3–6 *P ≤ 0.05 **P ≤ 0.01, ***P ≤ 0.001 ****P ≤ 0.0001. Error bars indicate mean ± SEM

Eef2 is fully active in its dephosphorylated state and is inhibited following phosphorylation by Eef2 kinase (Eef2K) [45]. In contrast, activating the mTOR pathway reduces Eef2 phosphorylation by inhibiting EF2K, and previous research has shown Eef2 to be upregulated during cardiac hypertrophy [10]. Our RIP-Seq data showed that Eef2 mRNA is bound to Ybx1, and it is down-regulated on a protein level after Ybx1 knockdown, whereas it remains unchanged at the mRNA level. Immunoblotting confirmed a 50% reduction in Eef2 protein levels after Ybx1 knockdown (Fig. 6A, B). RT-PCR showed that mRNA levels of Eef2 remain unchanged after Ybx1 knockdown in NRCMs, suggesting a translational control of Eef2 by Ybx1 (Fig. 6C). To validate the role of Eef2 in cardiomyocytes with decreased levels of Ybx1, we stimulated Eef2 activity in NRCMs after Ybx1 knockdown using the Eef2K inhibitor A-484954 [23, 24]. Treatment of NRCMs with A-484954, in control conditions, for 24 h results in a 50% reduction of phosphorylation levels of Eef2 compared to total Eef2, which was confirmed by immunoblots (Fig. 6D, E). Inhibition of Eef2K dephosphorylated and activated Eef2, which increased protein synthesis (Fig. 6F). In addition, we also performed rescue experiments, and the Eef2 activation after treatment with the Eef2K inhibitor was able to rescue the reduction in cell size in NRCMs after Ybx1 knockdown (Fig. 6G, H, Supplementary Fig. 2A, B). To study the protein synthesis in NRCMs under similar conditions, a puromycin assay was performed after knocking-down Ybx1 an treating NRCMs with an A-484954 for 24 h. As shown before, Ybx1 knockdown reduced puromycin incorporation, but the Eef2K inhibitor rescued the puromycin incorporation to normal conditions in Ybx1 knockdown NRCMs (Fig. 6I, J, Supplementary Fig. 2C, D). In conclusion, activation of Eef2 activity using A-484954 restored the reduction of overall protein synthesis in Ybx1 knockdown NRCMs cells.

**Fig. 7:**
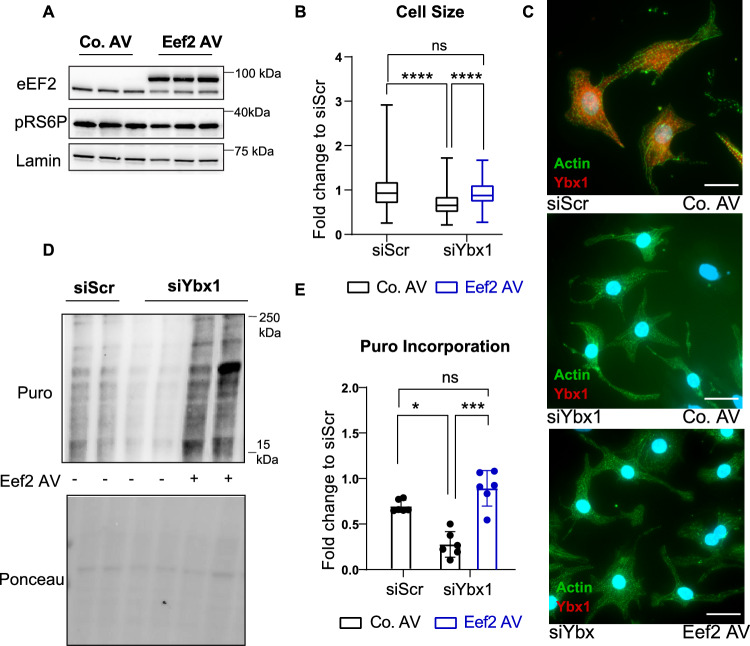
Eef2 overexpression increases cell size and protein translation in NRCMs. (**A**) Representative immunoblots of Eef2 overexpression (Eef2 AV) in NRCMs. The overexpressed Eef2 has a V5 tag and, therefore, runs higher than the endogenous levels. Lamin B was used as a housekeeping protein. (**B**) Quantification of cell size measurements after Ybx-1 knockdown and overexpression of control adenovirus (Co. AV) or Eef2 adenovirus (Eef2 AV). Analyzed by One-way ANOVA. *n* > 150 cells from *n* = 3 independent experiments. (**C**) Immunofluorescence staining of neonatal rat cardiomyocytes for Ybx1 (red), sarcomeric actin (green) and nuclei (blue) after knockdown of Ybx1 or scramble and Eef2 overexpression (100 uM). Scale bar 20 μm. (**D**) Representative immunoblot of puromycin incorporation in NRCMs with Eef2 overexpression after Ybx1 knockdown. Ponceau is used as a loading control. (**E**) Quantification of Puromycin incorporation in NRCMs after Ybx1 knockdown and Eef2 overexpression (Eef2AV). One-way ANOVA, *n* = 3–6 *P ≤ 0.05 **P ≤ 0.01, ***P ≤ 0.001 ****P ≤ 0.0001. Error bars indicate mean ± SEM

In addition to the pharmacological activation of Eef2 by A-484954, Eef2 levels were increased using an adenoviral vector. Immunoblots confirmed successful overexpression of Eef2 (Fig. 7A). Furthermore, immunofluorescence was performed on NRCMs after Ybx1 knockdown, followed by overexpression of Eef2 (Fig. 7B, C, Supplementary Fig. 3A, B), to confirm the rescue of cell size after Ybx1 knockdown. Again, decreased cell size after Ybx1 knockdown depended on Eef2 levels as increased Eef2 expression restored NRCMs size after Ybx1 knockdown. Puromycin assay was performed on NRCMs, after knockdown of Ybx1 and overexpression of Eef2, to examine the effect of Eef2 overexpression on overall protein translation. Overexpressing Eef2 was able to rescue the reduction in puromycin incorporation after Ybx1 knockdown (Fig. 7D, E, Supplementary Fig. 3C, D). Since Ybx1 expression was recently shown to regulate NRCMs proliferation, we also tested whether overexpression of Eef2 can rescue NRCM proliferation after Ybx1 knockdown. As previously shown, knockdown of Ybx1 decreased proliferation of NRCMs assessed by incorporation of 5-ethynyl-2'-deoxyuridine (EdU) into newly synthesized DNA as well as by staining of NRCMs for the proliferation marker Ki67. Restoration of Eef2 levels partly rescued the defect in proliferation, suggesting that reduced levels of Eef2 are causal for the decreased NRCMs size as well as for the decreased proliferation after Ybx1 depletion (Supplementary Fig. 3E–G).

### Ybx1 affects pathological cardiomyocyte growth in vivo

**Fig. 8:**
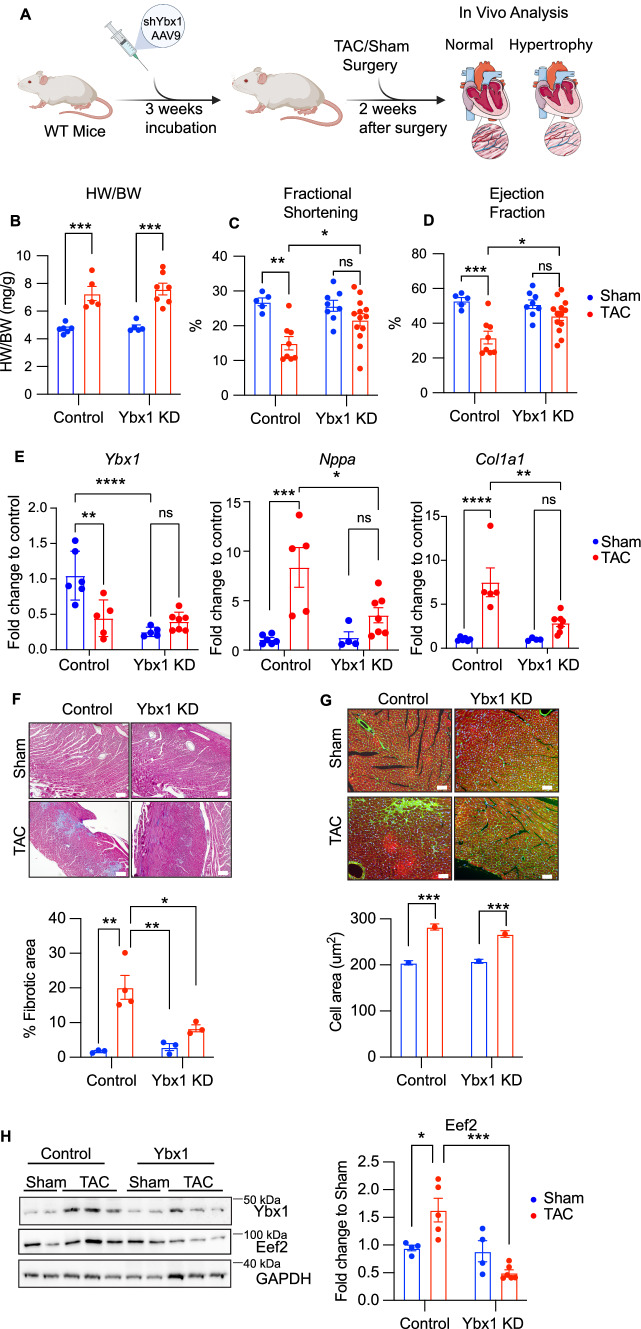
Ybx1 knockdown in vivo preserves heart function 2 weeks after TAC. (**A**) Experimental design for the in vivo experiments after Ybx1 KD. 2-month-old mice were injected with AAV9 shRNA against Ybx1 or control and then 3 weeks later TAC surgeries were performed. 2 weeks after the TAC or sham, echocardiography was performed, and mice were sacrificed for further analysis. Illustration created with Biorender.com (**B**) Heart weight to body weight (HW/BW) ratio, (**C**) Fractional Shortening and (**D**) Ejection fraction for sham and TAC mice in control or Ybx1 knockdown (Ybx1 KD) mice 2 weeks after TAC. *n* ≥ 5 (**E**) *Ybx1*, *Nppa*, *Col1a1* mRNA levels in vivo after TAC surgery in control and Ybx1 KD mice. (**F**) Representative Masson Trichrome staining of heart sections from WT and KD mice after Sham or TAC surgery. White line represents 200 µm and quantification of fibrotic areas. (**G**) Immunohistochemistry staining of heart sections from WT and KO mice after Sham or TAC surgery, stained for membrane proteoglycans by WGA (green), actinin (red) and for nuclei by DAPI (blue) to visualize the cross-sectional area of the cardiomyocytes and quantification of the cross-sectional cell surface area. (**H**) Representative immunoblot and quantification of Ybx1 in adult mouse left ventricle samples 2 weeks after TAC in control and Ybx1 knockdown mice. Analyzed by one-way ANOVA *P ≤ 0.05 **P ≤ 0.01, ***P ≤ 0.001. Error bars indicate mean ± SEM

Finally, to analyze the role of Ybx1 in vivo*,* we used an AAV9 vector with shRNA targeting Ybx1 to deplete Ybx1 (Ybx1 KD) in mouse hearts. Successful KD of Ybx1 in cardiomyocytes was confirmed by immunofluorescence in paraffin-embedded sections as well as in immunoblots of isolated cardiomyocytes from control or AAV-shRNA treated mice 3 weeks after injection (Supplementary Fig. 4A–B). Accordingly, 3 weeks after the injection, TAC surgeries were performed (Fig. 8A). Cardiac function 2 weeks after TAC surgery was assessed by ejection fraction, fractional shortening, and heart weight to body weight ratio. Heart weight to body weight ratio was increased in control animals and Ybx1 KD mice 2 weeks after TAC surgery. No significant difference in the HW/BW ratio between control and Ybx1 knockdown mice was observed after TAC surgery (Fig. 8B). Still, echocardiography's analysis of the heart function 2 weeks after TAC surgery showed that Ybx1 KD preserved fractional shortening and ejection fraction after TAC surgeries compared to control mice, which demonstrated decreased heart function (Fig. 8C, D, Table 1). Furthermore, Ybx1 knockdown led to a block in upregulation of the fetal gene markers generally upregulated in the adult heart during stress, such as *Nppa* and fibrosis markers, such as *Col1a1* (Fig. 8E). In line with decreased molecular markers of fibrosis, quantification of the fibrotic area in sections showed decreased fibrosis after Ybx1 depletion in response to TAC compared to control animals (Fig. 8F), whereas cardiomyocyte cell area was increased in TAC challenged mice (Fig. 8G). Those in vivo data indicate that therapeutic silencing of Ybx1 protects cardiac function after TAC surgery for 2 weeks, which is associated with decreased tissue fibrosis. Ybx1 reduction by shRNA in vivo at this timepoint was confirmed by immunoblotting from whole heart lysates (Fig. 8H). Ybx1 protein levels were increased after TAC surgeries, but this induction was blocked in mice treated with shRNA against Ybx1. Immunoblotting for Eef2 in Ybx1 KD mice hearts showed a reduction in Eef2 levels on a protein level, suggesting that Ybx1 is required for increased Eef2 expression during pressure overload.

**Table 1:** Characterization of mice after sham or TAC by echocardiography 4 wk after surgery

4 wk after surgery	Sham		TAC		
	Control	YB1 KD	Control		YB1 KD
	n = 5	n = 8	n = 8		n = 13
LVAW, mm	0.87 ± 0.08	0.96 ± 0.03	1.12 ± 0.03*		1.14 ± 0.14
LVPW,mm	1.09 ± 0.1	1.01 ± 0.07	1.34 ± 0.07*		1.33 ± 0.16
LVID, mm	3.66 ± 0.12	3.89 ± 0.19	4.18 ± 0.34*		4.01 ± 0.12^#^
EF, %	53.48 ± 3.31	48.01 ± 2.41	34.41 ± 6.13*		42.58 ± 4.46^#^
FS, %	27.23 ± 2.27	26.23 ± 1.36	16.55 ± 3.19*		21.06 ± 2.33^#^
HR, bpm	485 ± 35	423 ± 25	469 ± 29		486 ± 23

*EF* %, ejection fraction, *FS* %, fractional shortening, *HR* bpm, heart rate, *LVID* mm, left ventricular end-diastolic dimensions, *LVPW* mm, left ventricular posterior wall dimensions, *LVAW* mm, left ventricular anterior wall dimensions, *n* number of mice analyzed

*P < 0.05 vs. control sham; ^#^*P* < 0.05 vs. control TAC

We also wanted to test the effect of the Ybx1 knockdown in vivo after 4 weeks of TAC. Immunoblots were used to confirm the Ybx1 reduction and surprisingly Ybx1 levels were upregulated in the Ybx1 KD mice compared to the control group 4 weeks after TAC surgery (Supplementary Fig. 4C, D). In line with the previous finding, Ybx1 expression significantly increase after TAC both in control group but also in the Ybx1 KD mice. HW/BW ratio as well as cardiac function or molecular markers are unchanged in Ybx1 KD mice compared to controls. (Supplementary Fig. 4E–H). However, mRNA levels of Ybx1 in the KD mice were reduced significantly as compared to the control mice (Supplementary Fig. 4 H). Ybx1 mRNA levels after TAC surgery were unchanged, which again suggests that the increase in Ybx1 protein levels is regulated independent from the mRNA levels. Those experiments suggest that the upregulation of Ybx1 on the translational level in mice 4 weeks after TAC could be due to the self-regulating ability of Ybx1.

## Discussion

Several hundred RBPs that post-transcriptionally regulate RNA fate have been discovered in cardiomyocytes, but only a few have been comprehensively characterized and investigated to date [9, 28]. Transport, localization, regulation of mRNA stability, and translational control by RBPs allow for direct control of the spatial and temporal profiles of gene products. Such interactions can occur at the 5' UTR, the main ORF (CDS), or the 3' UTR of target mRNAs. Identifying proteins that associate with mRNA in cardiomyocytes by RIC has enhanced our understanding of how specific mRNA–Protein interactions regulate gene expression and cardiac function [28, 41].

In addition to RBPs, others and our work showed that the kinase mechanistic target of rapamycin mTOR directly controls mRNA translation and protein synthesis [4, 18, 31, 38]. Mechanistically, mTOR promotes the translation of a specific subset of mRNAs by phosphorylation of the eIF4E binding proteins (4E-BPs). mTOR sensitive transcripts often contain a terminal oligopyrimidines motif (TOP or TOP-like motif) in the 5' UTR and mTOR-dependent transcripts are predominantly regulated on the translational level [34]. Thereby, activation of mTORC1 in response to growth signals in turn leads to altered expression of specific proteins through direct translational control.

In our study, we identified Ybx1 as an RNA binding protein in cardiomyocytes that is involved in pathological cardiac growth and is translationally controlled by mTOR. The role of Ybx1 in cancer has been studied before, where Ybx1 is involved in the proliferation of tumor cells and is also considered a prognostic marker for malignant tumor growth [15, 30, 39]. Moreover, Ybx1 expression was recently linked to cardiomyocyte proliferation downstream of the cardiomyocyte enriched circularRNA CircNfix [20]. In this study, Ybx1 expression was dependent on proteasomal degradation upon binding to CircNfix and knockdown of Ybx1 decreased cardiomyocyte proliferation, suggesting that Ybx1 has a broad role in cardiomyocyte biology and pathophysiology not limited to growth control. Our results suggest that Ybx1 is directly controlled by mTOR in cardiomyocytes and specific pharmacological inhibition of mTOR kinase activity decreases the translation of Ybx1. The dependence of Ybx-1 on mTOR is most likely mediated by the presence of a TOP-like motif in the 5'UTR. We also showed that Ybx1 controls NRCMs cell size as well as proliferation via regulation of protein synthesis. Knocking down Ybx1 in NRCMs resulted in smaller cells, and this effect was preserved after stimulation with PE during in vitro cardiac hypertrophy. Ybx1 depletion prevents cellular growth in vitro by inhibiting protein synthesis, suggesting that translational controlled expression of Ybx1 is necessary to increase protein synthesis which is required for increased cellular mass. Mechanistically we identified around 700 transcripts that bound to Ybx1 during cardiac hypertrophy, and among this data set, we selected 7 targets that were also translational regulated by Ybx1 in cardiomyocytes. Since Ybx1 depletion was associated with a substantial reduction in overall mRNA translation, we focused our mechanistic follow-up studies on the role of Eef2 downstream of Ybx1. Mechanistically, we could causally link Eef2 expression to the observed phenotype after Ybx1 depletion both in vitro and in vivo. In line with a previous study [20], Ybx1 knockdown also decreased NRCM proliferation, which was again dependent on Eef2 protein levels.

Eef2 is on one hand upregulated in mice during cardiac remodeling, but also activated by dephosphorylation [6, 48]. Activity of *Eef2* can be inhibited by phosphorylation of Thr56 by Eef2k, also known as Ca^2+^ and calmodulin-dependent protein kinase [37]. Eef2 phosphorylation results in elongation inhibition and, thus, a decrease in global translation. In contrast, mTORC1 blocks Eef2K, resulting in enhanced elongation during cellular growth. This suggests a strong interplay between mTORC1 and translational elongation in eukaryotic cells.

Our work now further connects translationally controlled Eef2 expression with the regulation of protein synthesis, which is required for pathological cardiac hypertrophy. Translation of mRNAs is a key step in the regulation gene expression, and regulation of this process allows the immediate and direct adaptation of protein levels, independent of mRNA transcription [5]. The initiation phase of protein synthesis is considered a key regulator in the process. Elongation is a tightly regulated process as well, but compared to initiation, it has been relatively less explored in cardiac dysfunction. Increased protein levels or enhanced activity of Eef2 might be itself additional driver of cardiomyocyte growth [32, 36, 44]. Intriguingly inhibition of protein synthesis at the level of translational elongation has been shown to prevent pathological hypertrophy and improve cardiac function [6, 16, 17].

In cardiomyocytes, Ca^2+^ cycling, contraction and protein synthesis consume high proportion of cellular energy*.* Elevated protein synthesis might increase energy consumption which could subsequently negatively affect energy availability for contraction during pathological cardiac remodeling [25]. Alternatively, an increase in overall protein synthesis could lead to enhanced misfolded proteins and endoplasmic reticulum stress, which is clearly linked to cardiac dysfunction during pathological remodeling [12]. Follow-up studies are now needed to understand the causal role of Eef2 activity for cardiac dysfunction and the regulation of protein synthesis.

Finally, we tested the hypothesis that Ybx1 depletion is protective against pathological remodeling in vivo. Ybx1 was depleted in mice hearts in vivo using an shRNA targeting Ybx1 with an AAV9. TAC surgery was performed 3 weeks after AAV9 injections and cardiac function was preserved in Ybx1 knockdown mice after TAC. Interestingly, Ybx1 knockdown also blunted the increase in hypertrophic markers *Nppa* and *Col1a1 as well as overall fibrosis*. The role of in vivo has not been studied to date, and this study sheds light on the protective nature of reducing Ybx1 levels in vivo in cardiac hypertrophy. Although this approach resulted in reduced mRNA levels in Ybx1 KD mice at different timepoints after TAC, we observed a strong upregulation of Ybx1 protein levels 4 weeks after TAC surgery, which again suggests the translational regulation of Ybx1 in response to TAC. In line we could only see therapeutic benefits after Ybx1 KD up to 2 weeks after TAC when proteins levels were reduced compared to control mice. Genetic approaches in cell type specific KO mouse models would be needed to test whether long term reduction of Ybx1 in cardiomyocytes is protective against pressure overload.

In conclusion, activation of mTORC1 in response to growth signals increases Ybx1 expression which links signaling cascades to post-transcriptional regulation of gene expression. However, it is unclear whether those findings are relevant to human hearts, but Ybx1 expression is decreased in human heart failure data sets, suggesting that Ybx1 expression is dysregulated in human hearts as well [1, 26]. Understanding the complexity of gene regulation by RBPs during stress will allow us to choose specific RPBs that will aid in modifying the diseased translatome. With the help of existing technologies, such as RNA interference, we can target and degrade specific mRNAs. Interestingly, recent research has shown that RNA-specific targeted CRISPR might be used to manipulate mRNAs, so that the mRNA cleaving activity is specific, and no collateral mRNA molecules are targeted [1, 26]. This knowledge provides the exciting possibility of modifying the translatome by altering RBP expression, making it conceivable to direct molecular pathways involved in disease pathogenesis. The dynamic nature of translational regulation and the study of the proteins involved in the regulation will lead to an improved understanding of biology and pathophysiology and hopefully also to the development of novel therapeutic options.

### Supplementary Information

Below is the link to the electronic supplementary material.

Supplementary file1 (PDF 5943 KB)

Supplementary file2 (XLSX 952 KB)

Supplementary file3 (XLSX 1823 KB)

## Data Availability

The datasets generated and/or analyses performed in the present study are available from the corresponding author on reasonable request.
