# Supplementary material for: Translational control of Ybx1 expression regulates cardiac function in response to pressure overload in vivo
Source: Basic Res Cardiol. 2023 Jun 28;118(1):25. doi: 10.1007/s00395-023-00996-1 (PMC10307726; doi:10.1007/s00395-023-00996-1)
Supplement: Supplementary file 1 — Supplementary file1 (PDF 5943 KB) [file 395_2023_996_MOESM1_ESM.pdf]

Supplementary data

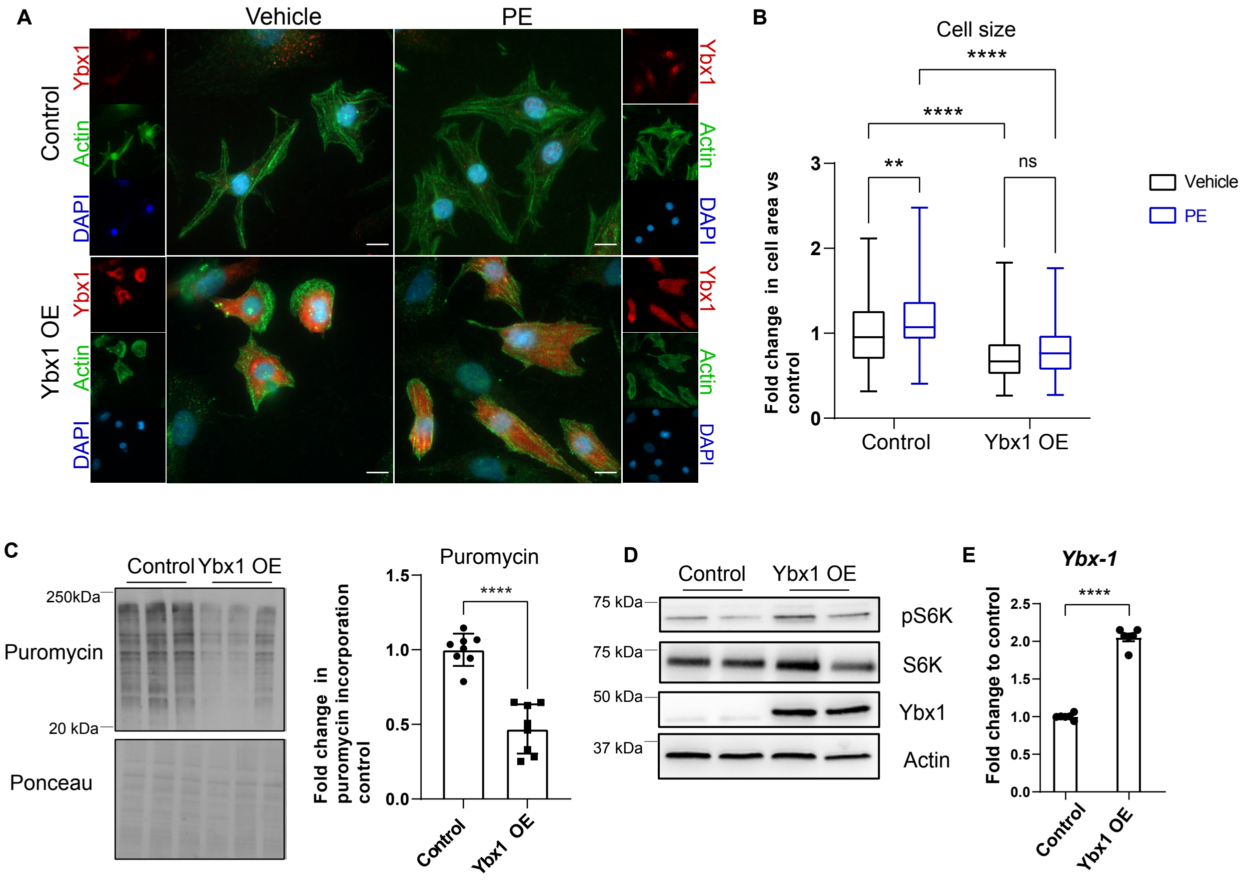

Supplementary Figure 1

(A) Immunofluorescence staining of neonatal rat cardiomyocytes for Ybx1 (red), sarcomeric actin (green) and nuclei (blue) after overexpression of Ybx1 (Ybx1 OE) or control Virus (Control) with/without PE (50uM) treatment for 24hrs. Scale bar 20μm. **(B)** Quantification of cell size measurements. Analysed by One-way ANOVA. n>150 cells from n=3 independent experiments. **(C)** Representative immunoblot and quantification of Puromycin incorporation in NRCM after adeno viral vector expression with control virus (Control) or Ybx1 overexpression (Ybx1 OE). Ponceau staining was used as a gel loading control. n=4-6 per group. **(D)** Representative immunoblot of Ybx1 protein levels in NRCM Ybx1 overexpression. Actin is used as a control. n = 6 independent experiments. **(E)** Quantification of *Ybx1* mRNA levels in NRCM after overexpression of Ybx1 in NRCM t-test, ** - P ≤ 0.01, **** - P ≤ 0.0001. Error bars indicate mean ± SEM.

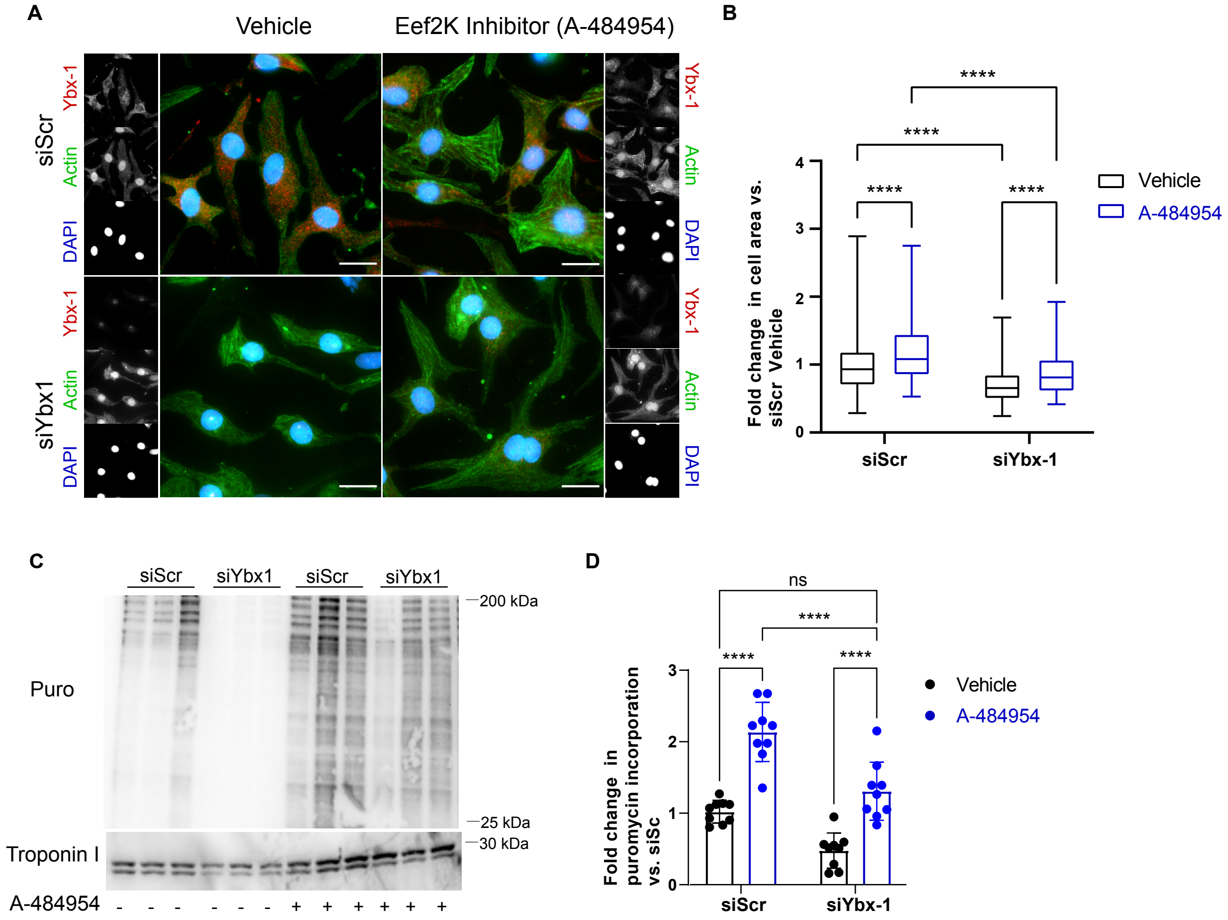

Supplementary Figure 2: eEF2K inhibitor increases cell size and protein translation in NRCMs **(A)** Immunofluorescence staining of neonatal rat cardiomyocytes for Ybx1 (red), sarcomeric actin (green) and nuclei (blue) after knockdown of Ybx1 or scramble and with/without eEF2 Kinase inhibitor (100 uM). Scale bar 20μm. **(B)** Quantification of cell size measurements. Analysed by One-way ANOVA. n>150 cells from n=3 independent experiments. **(C)** Representative immunoblots of puromycin incorporation in NRCM treated with eEF2 inhibitor for 24 hours after Ybx1 knockdown. Troponin is used as a loading control. **(D)** Quantification of Puromycin incorporation in NRCM after Ybx1 knockdown and treatment with eEF2K inhibitor. One-way ANOVA, n=3-6, **** - $P \leq 0.0001$. Error bars indicate mean ± SEM

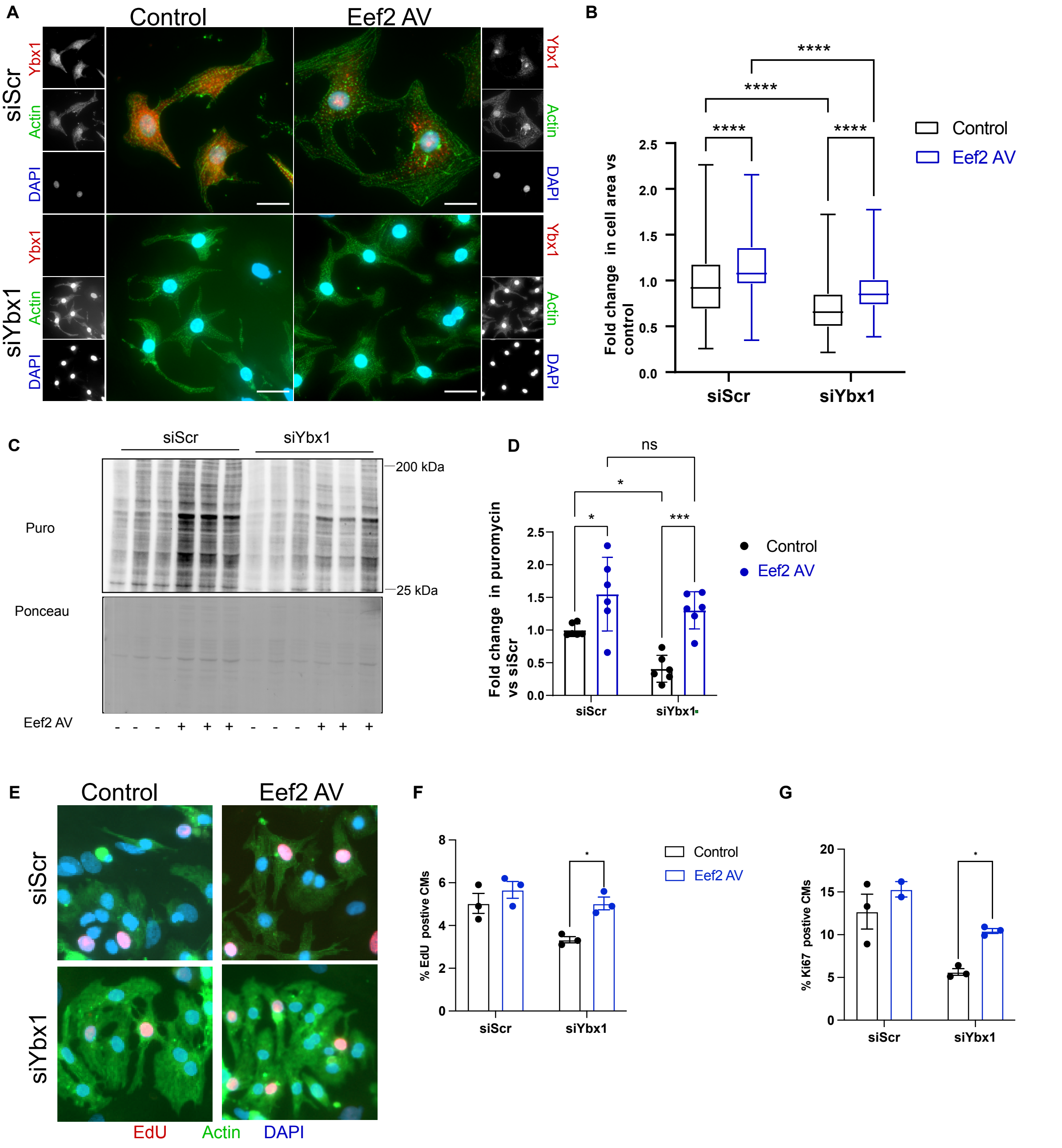

Supplementary Figure 3: eEF2 overexpression increases cell size and protein translation in NRCMs

(A) Immunofluorescence staining of neonatal rat cardiomyocytes for Ybx1 (red), sarcomeric actin (green) and nuclei (blue) after knockdown of Ybx1 or scramble and eEF2 overexpression (100 uM). Scale bar 20μm. **(B)** Quantification of cell size measurements. Analyzed by One-way ANOVA. n>150 cells from n=3 independent experiments. **C)** Representative immunoblots of puromycin incorporation in NRCM with eEF2 overexpression after Ybx1 knockdown. Ponceau is used as a loading control. **(D)** Quantification of Puromycin incorporation in NRCM after Ybx1 knockdown and eEF2 overexpression (eEF2AV). One-way ANOVA, n=3-6 * - $P \leq 0.05$, *** - $P \leq 0.001$ **** - $P \leq 0.0001$. Error bars indicate mean ± SEM.**(E)** Representative immunofluorescence of EdU incorporation (red) sarcomeric actin (green) and nuclei (blue) after knockdown of Ybx1 or scramble and eEF2 overexpression. **(F)** Quantification of EdU incorporation (EdU positive cells) in NRCMs after Ybx1 knockdown and eEF2 overexpression (eEF2AV). One-way ANOVA, n=3 independent experiments - *$P \leq 0.05$. Error bars

indicate mean ± SEM. **(G)** Quantification of Ki67 positive cells in NRCMs after Ybx1 knockdown and eEF2 overexpression (eEF2AV). One-way ANOVA, n=3 independent experiments - *$P \leq 0.05$. Error bars indicate mean ± SEM

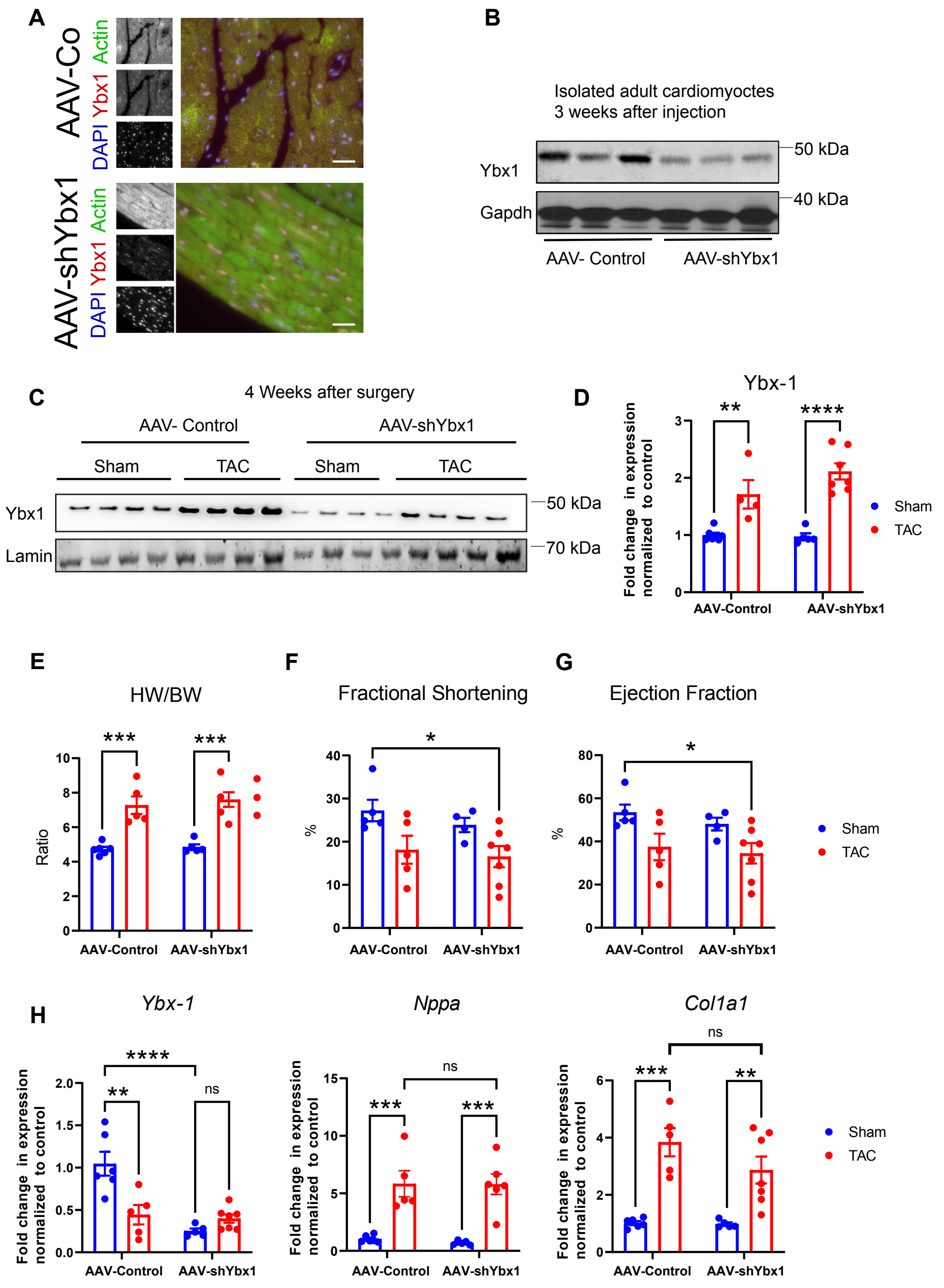

Supplementary Figure 4: Ybx1 knockdown *in vivo* 4 weeks after TAC

(A) Staining of heart sections from WT and KD mice stained for Ybx1 (green), actinin (red) and for nuclei by DAPI (blue) to visualize the KD of Ybx1 in cardiomyocytes. Scale bar 20um **(B)** Representative immunoblot and quantification of Ybx1 in isolated adult cardiomyocytes in control and Ybx1 knockdown mice. **(C)** Representatives immunoblots and **(D)** quantification of Ybx1 in adult mouse left ventricle samples 4 weeks after TAC in control and Ybx1 knockdown mice. **(E)** Heart Weight to Body Weight (HW/BW) ratio, **(F)** Fractional Shortening and **(G)** Ejection fraction for sham and TAC mice in control or Ybx1 knockdown (Ybx1 KD) mice 2 weeks after TAC. n ≥ 4 for each group. **(H)** *Ybx1*, *Nppa*, *Col1a1* mRNA levels *in vivo* after TAC surgery in control and Ybx1 KD mice. n ≥ 5 Analyzed by one-way ANOVA * - $P \leq 0.05$ ** - $P \leq 0.01$, *** - $P \leq 0.001$, **** - $P \leq 0.0001$. Error bars indicate mean ± SEM
